# The prevalence of potentially traumatic events in the seventh survey of the population-based Tromsø study (Tromsø 7)

**DOI:** 10.1177/14034948211051511

**Published:** 2021-10-20

**Authors:** Jens C. Thimm, Kamilla Rognmo, Marte Rye, Anna Margrete Flåm, Eva Therese Næss, Ingunn Skre, Catharina E. A. Wang

**Affiliations:** 1 Centre for Crisis Psychology, University of Bergen, Norway; 2 Department of Psychology, UiT The Arctic University of Norway, Norway; 3 Regional Centre for Child and Youth Mental Health and Child Welfare, UiT The Arctic University of Norway, Norway

**Keywords:** Potentially traumatic live events, prevalence, population-based study, Tromsø study

## Abstract

*Aims:* Potentially traumatic events (PTEs) can have detrimental consequences for an individual’s physical and mental health. Exposure to PTEs is therefore increasingly assessed in population-based studies. Consistent with this trend, the most recent wave of the longitudinal population-based Tromsø study (Tromsø 7) in Northern Norway included a list of PTEs. The aim of the present study was to describe the prevalence of PTEs in the sample and examine demographic correlates of reported PTE exposure in this group. *Methods:* In Tromsø 7, a total of 21,083 participants aged ⩾40 years (52.5% female, mean age 57.3 years) were asked about exposure to nine PTEs that occurred in childhood, in adulthood and in the previous year. Differences between demographic groups in exposure to PTEs were examined using chi-square tests and logistic regression analyses. *Results:* Overall, 67% of the participants reported at least one PTE across the three time intervals. A life-threatening illness or serious accident of a loved one (36.8%) or of the respondent (24.0%) and bullying (21.5%) were the most frequently reported PTEs. Female sex, younger age, indigenous or immigrant ethnicity and higher education were associated with an increased likelihood of reporting at least one PTE. Group differences with respect to specific PTEs were observed. ***Conclusions:* The experience of PTEs is common among the participants in the Tromsø 7 study. The current study lays the foundation for further research into the associations between PTEs and physical and mental health within the Tromsø study.**

## Introduction

The detrimental effect of highly stressful and potentially traumatic events (PTEs) on a person’s well-being and health has been recognised since ancient times [1] Although there is no generally accepted definition of PTEs [2], most approaches to stressful life events include a defining element of threat or harm [3]. For example, exposure to death or threatened death, serious injury or illness, or sexual violence are generally considered PTEs. Estimates on the prevalence of PTEs in the general population vary between and within countries, depending on sampling and the number of PTEs included in the investigations [4]. For example, in a study conducted in 24 countries, 70.4% of the respondents experienced at least one of the 29 PTEs assessed in the survey, ranging from 28.6% in Bulgaria to 84.6% in Ukraine [5].

Few studies have investigated the prevalence of PTEs in adults in Norway, and, as with the international results, the findings diverge. In a sample of young adults aged 19–36 years (*N*=2794), 26.5% of the participants reported that they had personally experienced or witnessed at least one of eight PTEs included in the investigation [6]. In another study, 25.9% of the men and 20.6% of the women in the general population aged ⩾18 years (*N*=1634) reported exposure to at least one of 11 PTEs [7]. Recently, an incidence of PTEs in men and women of 85% and 86%, respectively, was found when 17 PTEs were measured in the general population (*N*=1792, age range 18–94 years) [8]. Findings from these studies suggest that women report more exposure to sexual assault and abuse than men do [6,8]. It has been further found that indigenous Sami and Kven Norwegian citizens more frequently reported being victims of bullying and ethnic discrimination compared to non-Sami Norwegian citizens [9].

Numerous investigations have found associations between PTEs and a variety of physical health problems or illnesses [10,11] and mental disorders [6,12]. A dose–response relationship has been observed with an increased risk of developing health problems with a higher number of PTEs [13]. Furthermore, findings suggest differential effects of PTEs depending on the type of PTE and the age of exposure, with childhood PTEs having a more significant impact than PTEs in adulthood [13,14]. Changes in the hypothalamic–pituitary–adrenal axis and inflammatory processes have been proposed as mediating mechanisms between trauma exposure and physical and mental illness [15].

Because concerns about potential harmful effects of asking people about PTEs have been shown to be unfounded [16], and due to the significant impact that PTEs can have on a person’s mental and physical health, assessments of PTEs are more often included in large health studies of the general population [17]. The Tromsø study [18] is a longitudinal population-based study of residents of the municipality of Tromsø in Northern Norway. The Tromsø study was conducted for the first time in 1974 to investigate causes of high mortality due to cardiovascular diseases in men at that time [18]. In subsequent waves of the Tromsø study, women were included, and additional diseases and conditions were examined. However, in order to be able to investigate the possible connection between traumatic life events and a number of outcome measures related to physical and mental health and illness, collecting data on the prevalence of PTEs among participants in the Tromsø survey is important. Accordingly, in the most recent wave (Tromsø 7), it was decided to assess several PTEs, including sexual abuse, serious illness, bullying and painful medical treatment. Although few previous studies have regarded bullying as a PTE, exposure to bullying was included in the Tromsø 7 study due to demonstrated relationships between bullying and poorer mental and/or physical health [19]. In Tromsø 7, the participants were asked about exposure to PTEs before the age of 18, after the age of 18 and in the previous year. This makes it possible to investigate the role of PTEs in childhood and in adulthood for mental and physical health in addition to the effects of exposure to multiple PTEs, which were not examined in the present study but can be analysed in future research.

The aim of the present study was to examine the prevalence of PTEs and the associations of PTEs with demographic characteristics in the Tromsø 7 population to facilitate research into the associations between PTEs and physical and mental health within the Tromsø study. In addition, due to the large sample size of the Tromsø 7 study and the assessment of bullying as a PTE – which has not been included in previous Norwegian studies – the investigation of the frequency of PTEs in Tromsø 7 will contribute to increased knowledge about PTEs in Norway.

## Method

### Participants

In Tromsø 7 (2015–2016), all residents aged ⩾40 years were invited to answer questions about their health and lifestyle and to undergo physical examinations. A total of 21,083 participated (64.7% of those invited), 11,074 (52.5%) of whom were female. The mean age for the entire sample was 57.3 years (*SD*=11.4 years, range 40–99 years). Further demographic characteristics of the sample are shown separately for women and men in Table I.

**Table I.** Demographic characteristics of the study sample (*N*=21,083).

	Women (*N*=11,074; 52.5%)	Men (*N*=10,009; 47.5%)
Age (years), *M* (*SD*)	57.2 (11.5)	57.4 (11.4)
Living with a spouse/partner	7403 (72.3%)	7880 (81.6%)
Ethnicity^a^		
Norwegian	10,363 (93.6%)	9472 (94.6%)
Sami	323 (2.9%)	257 (2.6%)
Kven	236 (2.1%)	164 (1.6%)
Other than Norwegian, Sami or Kven	496 (4.5%)	388 (3.9%)
Education		
Primary/partial secondary education (up to 10 years of schooling)	2617 (24.1%)	2179 (22.2%)
Upper secondary education (a minimum of 3 years)	2759 (25.3%)	2997 (30.5%)
Tertiary education, short (college/university <4 years)	1917 (17.6%)	2091 (21.3%)
Tertiary education, long (college/university ⩾4 years)	3581 (32.9%)	2564 (26.1%)
Household income		
<150,000 NOK	134 (1.3%)	76 (0.8%)
150,000–250,000 NOK	635 (6.1%)	355 (3.6%)
251,000–350,000 NOK	911 (8.7%)	528 (5.4%)
351,000–450,000 NOK	1120 (10.8%)	786 (8.0%)
451,000–550,000 NOK	1319 (12.7%)	993 (10.2%)
551,000–750,000 NOK	1769 (17.0%)	1803 (18.5%)
>1,000,000 NOK	2271 (21.8%)	2470 (25.3%)
Occupation		
Works full time	5694 (52.2%)	6354 (64.6%)
Works part time	1248 (11.4%)	414 (4.2%)
Housekeeping	103 (0.9%)	29 (0.3%)
Retired	2526 (23.1%)	2261 (23.0%)
Disability benefit recipient/work assessment allowance	1239 (11.3%)	662 (6.7%)
Family income supplement	7 (0.1%)	18 (0.2%)
Unemployed	53 (0.4%)	84 (0.9%)
Student/military service	43 (0.4%)	17 (0.2%)

a Multiple responses possible.

NOK: Norwegian Krone (1 NOK≈US$0.11).

The present investigation was approved by the Regional Committee of Medical and Health Research Ethics (ref. 79060). The Norwegian Data Protection Service (NSD) was notified about the study (ref. 668477).

### Measures

Demographic and health information was collected with a four-page paper-and-pencil questionnaire sent along with the invitation letter and an additional online survey. The demographic variables age, sex, living with a spouse/partner, ethnicity, education, household income and occupation were used to describe the sample. The response categories are displayed in Table I. In the online survey, participants were asked if they had ever experienced one of the following events: (a) a life-threatening illness or a serious accident (e.g. fire, work accident or car accident); (b) violence (e.g. being hit, kicked, beaten, robbed or threatened with a firearm); (c) sexual abuse (i.e. sexual actions against one’s will); (d) bullying (e.g. been called negative things, marginalised, threatened or bullied by schoolmates, fellow students or co-workers over an extended period); (e) a loved one being exposed to violence or sexual abuse (e.g. hit, kicked, beaten, robbed or threatened with a firearm); (f) something else frightening, dangerous or violent (e.g. natural disaster, war, terror attack, being held captive); (g) severe grief after bereavement; (h) painful medical treatment when in hospital due to sickness or serious injury; (i) painful dental treatment; (j) a life-threatening illness or serious accident (e.g. fire, work accident or car accident) of a loved one; (k) childhood neglect (e.g. not having received the necessary food, clothing, protection and care/love from parents/caregivers). The response options for the events (a)-(j) were ‘no’, ‘yes, before the age of 18’, ‘yes, after the age of 18’ and ‘yes, in the previous year’. For childhood neglect (k), the response categories were ‘yes’ and ‘no’. The prevalence of (g), severe grief after bereavement, is reported in a separate publication [20]. The experience of painful dental treatment (i) is subject of another study. These PTEs are therefore not included in the present investigation.

### Analyses

The prevalence of PTEs was calculated for the entire sample and stratified for sex, age group, ethnicity and education. Age was categorised into five groups: 40–49 years, 50–59 years, 60–69 years, 70–79 years and 80–99 years. With respect to ethnicity, the following mutually exclusive groups were formed: Norwegian, dual Norwegian and Sami/Kven ethnicity, Sami/Kven and other ethnicities. The latter group was comprised of participants who had immigrant ethnicities only or in combination with Norwegian or the indigenous Sami and Kven ethnicities. Group differences in the exposure to PTEs were investigated using chi-square tests. Logistic regression analyses were conducted to further examine the associations between PTEs and demographic groups. The variables age group, education and ethnicity were dummy coded with the youngest age group, primary education and Norwegian ethnicity as reference categories, respectively. The relationships of sex, age group, ethnicity and education with the number of PTEs experienced were tested with binomial negative regressions due to a high number of zero counts. The analyses were performed in R v4.0.2 (R Foundation for Statistical Computing, Vienna, Austria) using the packages gmodels [21] and MASS [22].

## Results

### Overall prevalence of PTEs in the sample and sex differences

Overall, 67% of the participants reported exposure to at least one PTE before the age of 18, after the age of 18 or during the previous year. The most frequently reported PTE was a life-threatening illness or serious accident of a loved one (36.8%; see Table II). Twenty-nine per cent of the participants experienced one PTE, 17.8% two PTEs, 10% three PTEs, 5.2% four PTEs, 2.8% five PTEs and 5.8% six or more PTEs. The mean number of lifetime PTEs was 1.50 (standard deviation (*SD*)=1.67). More women (68.6%) than men (65.2%) experienced at least one PTE (*p*<0.001, odds ratio (OR)=1.17, 95% confidence interval (CI) 1.10–1.24). Women also reported significantly more PTEs (*M*=1.56, *SD*=1.71) than men did (*M*=1.43, *SD*=1.63, *p*<0.001). The prevalence of PTEs for the full sample and stratified by sex are shown in Table II. In total, 35.1% of the participants reported exposure to at least one PTE before the age of 18, 50.7% exposure after the age of 18 and 8.9% exposure during the previous year.

**Table II.** Prevalence of PTEs in the entire sample, stratified by sex.

PTEs	Lifetime	Before the age of 18				After the age of 18				Previous year			
	Overall	Overall	Women	Men	*p*	Overall	Women	Men	*P*	Overall	Women	Men	*p*
	*n* (%)	*n* (%)	*n* (%)	*n* (%)		*n* (%)	*n* (%)	*n* (%)		*n* (%)	*n* (%)	*n* (%)	
Serious illness or accident	4924 (24.0%)	1096 (5.3%)	514 (4.8%)	582 (6.0%)	<0.001	3653 (17.8%)	1625 (15.1%)	2028 (20.8%)	<0.001	389 (1.9%)	192 (1.8%)	197 (2.0%)	0.222
Violence	3203 (15.6%)	1213 (5.9%)	484 (4.5%)	729 (7.5%)	<0.001	2123 (10.4%)	956 (8.9%)	1167 (12.0%)	<0.001	93 (0.5%)	48 (0.4%)	45 (0.5%)	0.882
Sexual abuse	1986 (9.7%)	1502 (7.3%)	1205 (11.2%)	297 (3.0%)	<0.001	592 (2.9%)	542 (5.1%)	50 (0.5%)	<0.001	8 (0.0%)	7 (0.1%)	1 (0.0%)	^a^
Bullying	4412 (21.5%)	3316 (16.2%)	1661 (15.5%)	1655 (17.0%)	0.004	1169 (5.7%)	727 (6.8%)	442 (4.5%)	<0.001	295 (1.4%)	188 (1.8%)	107 (1.1%)	<0.001
Witnessed violence or sexual abuse	1925 (9.4%)	903 (4.4%)	526 (4.9%)	377 (3.9%)	<0.001	1040 (5.1%)	568 (5.3%)	472 (4.8%)	0.135	107 (0.5%)	66 (0.6%)	41 (0.4%)	0.053
Another frightening, dangerous or violent event	1715 (8.4%)	614 (3.0%)	361 (3.4%)	253 (2.6%)	0.001	1058 (5.2%)	413 (3.9%)	645 (6.6%)	<0.001	91 (0.4%)	53 (0.5%)	38 (0.4%)	0.260
Painful medical treatment	2235 (10.9%)	713 (3.5%)	395 (3.7%)	318 (3.3%)	0.099	1410 (6.9%)	799 (7.5%)	611 (6.3%)	<0.001	178 (0.9%)	98 (0.9%)	80 (0.8%)	0.471
Serious illness or accident of a loved one	7500 (36.8%)	890 (4.4%)	475 (4.5%)	415 (4.3%)	0.527	5983 (29.4%)	3518 (33.0%)	2465 (25.4%)	<0.001	1129 (5.5%)	659 (6.2%)	470 (4.8%)	<0.001
Childhood neglect	1417 (6.9%)	1417 (6.9%)	889 (8.2%)	528 (5.4%)	<0.001								
At least one PTE	13,867 (67.0%)	7258 (35.1%)	3938 (36.2%)	3320 (33.8%)	<0.001	10,439 (50.7%)	5670 (52.5%)	4769 (48.7%)	<0.001	1996 (8.9%)	1121 (10.4%)	875 (8.9%)	<0.001
Mean number PTEs (*SD*)	1.50 (1.67)	0.56 (0.97)	0.60 (1.01)	0.53 (0.92)	<0.001	0.83 (1.06)	0.85 (1.05)	0.81 (1.07)	0.006	0.11 (0.37)	0.12 (0.39)	0.10 (0.34)	<0.001

a Not calculated because at least one cell had expected frequencies <5.

PTE: potentially traumatic event.

Before the age of 18, the prevalence of specific PTEs ranged from 3.5% (painful medical treatment) to 16.2% (bullying). Significantly more women than men experienced sexual abuse, neglect, witnessing violence or sexual abuse and other frightening, dangerous or violent events before the age of 18 (ORs with 95% CIs are shown in Supplemental Table SI). Significantly more men than women reported exposure to violence, a life-threatening illness or a serious accident and bullying. After the age of 18, the prevalence of specific PTEs ranged from 2.9% (sexual abuse) to 29.4% (a life-threatening illness or a serious accident of a loved one). Significantly more women than men reported sexual abuse, bullying, a life-threatening illness or serious accident of a loved one and painful medical treatment in hospital. In contrast, significantly more men than women experienced other frightening, dangerous or violent events, life-threatening illness or a serious accident or violence. In the previous year, the prevalence of specific PTEs ranged from 0.1% (sexual abuse) to 5.5% (life-threatening illness or serious accident of a loved one). Significantly more women than men reported bullying and life-threatening illness or a serious accident of a loved one during the previous year.

### Prevalence of PTEs in different age groups

The prevalence of lifetime PTEs decreased significantly with increasing age. At least one PTE was reported by 72.1% of the participants in the 40–49 years age group compared to 59.6% in the 80–99 years age group (*p*<0.001). Participants aged 40–49 years reported on average significantly more PTEs (*M*=1.77, *SD*=1.88) than the other groups (50–59 years: *M*=1.65, *SD*=1.75; 60–69 years: *M*=1.25, *SD*=1.44; 70–79 years: *M*=1.06, *SD*=1.22; 80–99 years: *M*=1.13, *SD*=1.23; *p*<0.001). The prevalence of PTEs in the different age groups before the age of 18, after the age of 18 and during the previous year is displayed in Table III (see Supplemental Table SII for ORs). There were significant group differences for all PTEs before and after the age of 18 and for three PTEs during the previous year.

**Table III.** Prevalence of PTEs by age group.

	Before the age of 18						After the age of 18						Previous year					
	40–49	50–59	60–69	70–79	80–99	*p*	40–49	50–59	60–69	70–79	80–99	*P*	40–49	50–59	60–69	70–79	80–99	*p*
	*n* (%)	*n* (%)	*n* (%)	*n* (%)	*n* (%)		*n* (%)	*n* (%)	*n* (%)	*n* (%)	*n* (%)		*n* (%)	*n* (%)	*n* (%)	*n* (%)	*n* (%)	
Serious illness or accident	445 (7.1%)	343 (5.8%)	207 (4.1%)	77 (3.0%)	24 (3.5%)	<0.001	907 (14.4%)	1118 (18.9%)	963 (19.1%)	52^a^ (20.5%)	138 (20.1%)	<0.001	93 (1.5%)	116 (2.0%)	112 (2.2%)	57 (2.2%)	11 (1.6%)	0.032
Violence	583 (9.3%)	392 (6.6%)	181 (3.6%)	49 (1.9%)	8 (1.2%)	<0.001	889 (14.1%)	718 (12.2%)	402 (8.0%)	93 (3.6%)	21 (3.1%)	<0.001	51 (0.8%)	25 (0.4%)	16 (0.3%)	1 (0.0%)	0 (0.0%)	^a^
Sexual abuse	585 (9.3%)	505 (8.6%)	315 (6.2%)	84 (3.3%)	13 (1.9%)	<0.001	242 (3.8%)	189 (3.2%)	124 (2.5%)	32 (1.2%)	5 (0.7%)	<0.001	7 (0.1%)	1 (0.0%)	0 (0.0%)	0 (0.0%)	0 (0.0%)	^a^
Bullying	1480 (23.5%)	1063 (18.0%)	565 (11.2%)	175 (6.8%)	33 (4.8%)	<0.001	459 (7.3%)	381 (6.5%)	239 (4.7%)	77 (3.0%)	13 (1.9%)	<0.001	121 (1.9%)	121 (2.1%)	43 (0.9%)	10 (0.4%)	0 (0.0%)	<0.001
Witnessed violence or sexual abuse	437 (6.9%)	289 (4.9%)	139 (2.8%)	31 (1.2%)	7 (1.0%)	<0.001	409 (6.5%)	358 (6.1%)	187 (3.7%)	71 (2.8%)	15 (2.2%)	<0.001	51 (0.8%)	33 (0.6%)	14 (0.3%)	7 (0.3%)	2 (0.3%)	^a^
Another frightening, dangerous or violent event	117 (1.9%)	68 (1.2%)	60 (1.2%)	193 (7.5%)	176 (25.8%)	<0.001	369 (5.9%)	373 (6.3%)	209 (4.1%)	83 (3.2%)	24 (3.5%)	<0.001	39 (0.6%)	33 (0.6%)	9 (0.2%)	6 (0.2%)	4 (0.6%)	^a^
Painful medical treatment	235 (3.7%)	234 (4.0%)	171 (3.4%)	61 (2.4%)	12 (1.8%)	<0.001	465 (7.4%)	446 (7.6%)	303 (6.0%)	159 (6.2%)	37 (5.5%)	0.003	63 (1.0%)	52 (0.9%)	41 (0.8%)	15 (0.6%)	7 (1.0%)	0.401
Serious illness or accident of a loved one	394 (6.3%)	264 (4.5%)	164 (3.3%)	54 (2.1%)	14 (2.1%)	<0.001	1859 (29.6%)	1886 (32.1%)	1429 (28.5%)	650 (25.7%)	159 (24.0%)	<0.001	402 (6.4%)	335 (5.7%)	236 (4.7%)	133 (5.2%)	23 (3.5%)	<0.001
Childhood neglect	548 (8.7%)	473 (8.0%)	255 (5.0%)	107 (4.1%)	34 (4.8%)	<0.001												
At least one PTE	2814 (44.5%)	2206 (37.2%)	1389 (27.2%)	607 (23.2%)	242 (34.0%)	<0.001	3302 (52.3%)	3213 (54.2%)	2458 (48.4%)	1167 (45.1%)	299 (43.0%)	<0.001	697 (11.0%)	620 (10.5%)	420 (8.3%)	215 (8.3%)	44 (6.3%)	<0.001
Mean number PTEs (*SD*)	0.76 (1.10)	0.61 (1.02)	0.40 (0.88)	0.32 (0.69)	0.45 (0.75)	<0.001	0.88 (1.12)	0.92 (1.13)	0.76 (0.99)	0.65 (0.87)	0.61 (0.81)	<0.001	0.13 (0.42)	0.12 (0.38)	0.09 (0.33)	0.09 (0.30)	0.07 (0.27)	<0.001

a Not calculated because at least one cell had expected frequencies < 5.

For PTEs before the age of 18, the associations between PTEs and age were negative for all events, except for another frightening, dangerous or violent event, which was reported significantly more often by the oldest age groups compared to the youngest age group. For PTEs after the age of 18 and in the previous year, the likelihood of reporting a serious illness or accident increased with age but declined significantly for the other PTEs.

### Prevalence of PTEs and ethnicity

Overall, 66.1% of the Norwegian participants, 84% of the Sami/Kven participants, 79.4% of the participants with dual Norwegian and Sami/Kven identity and 74.7% of the participants with other ethnicities (*p*<0.001) had experienced at least one PTE during their lifetime. Participants with indigenous or immigrant ethnic identities experienced, on average, significantly more PTEs (Sami/Kven: *M*=2.51, *SD*=2.18; Norwegian–Sami/Kven: *M*=2.20, *SD*=2.00; other ethnicity: *M*=2.17, *SD*=2.24) than Norwegians (*M*=1.44, *SD*=1.61; *p*<0.001). Table IV presents the prevalence of PTEs before the age of 18, after the age of 18 and during the previous year in the different ethnic groups. Apart from a life-threatening illness or serious accident of a loved one before the age of 18, results showed significant differences between Norwegians, Sami/Kven, participants with dual Norwegian–Sami/Kven ethnic identity and participants with ethnicities other than Norwegian and/or Sami/Kven. Participants with ethnicity other than solely Norwegian reported considerably more exposure to the specific PTEs before and after the age of 18 than the Norwegian participants did (see Supplemental Table SIII for ORs). With respect to PTE exposure in the previous year, low prevalence rates in the four groups prevented statistical analyses for most PTEs. Therefore, the three non-Norwegian groups were combined into one group. Results showed that participants with ethnicity other than solely Norwegian were significantly (*p*<0.05) more likely to have experienced all assessed PTEs in the previous year except for witnessing violence or sexual abuse (*p*=0.059) than Norwegian participants. For sexual abuse, the counts were still too low for statistical analyses. ORs ranged from 1.27 (illness or accident of a loved one) to 2.37 (violence).

**Table IV.** Prevalence of PTEs by ethnicity.

	Before the age of 18					After the age of 18					Previous year				
	Norw.	Sami/Kven	Norw.-Sami/Kven	Other	*p*	Norw.	Sami/Kven	Norw.-Sami/Kven	Other	*p*	Norw.	Sami/Kven	Norw.-Sami/Kven	Other	*p*
	*n* (%)	*n* (%)	*n* (%)	*n* (%)		*n* (%)	*n* (%)	*n* (%)	*n* (%)		*n* (%)	*n* (%)	*n* (%)	*n* (%)	
Serious illness or accident	955 (5.1%)	21 (9.9%)	60 (9.7%)	59 (7.0%)	<0.001	3283 (17.5%)	45 (21.1%)	146 (23.7%)	164 (19.4%)	<0.001	337 (1.8%)	3 (1.4%)	20 (3.2%)	27 (3.2%)	^a^
Violence	1018 (5.4%)	27 (12.7%)	61 (9.9%)	103 (12.2%)	<0.001	1820 (9.7%)	49 (23.1%)	106 (17.2%)	143 (16.9%)	<0.001	76 (0.4%)	2 (0.9%)	4 (0.6%)	10 (1.2%)	^a^
Sexual abuse	1301 (6.9%)	27 (12.7%)	94 (15.2%)	77 (9.1%)	<0.001	491 (2.6%)	14 (6.6%)	39 (6.3%)	45 (5.3%)	<0.001	5 (0.0%)	0 (0.0%)	1 (0.2%)	2 (0.2%)	^a^
Bullying	2913 (15.5%)	60 (28.3%)	161 (26.1%)	176 (20.8%)	<0.001	980 (5.2%)	31 (14.6%)	66 (10.7%)	87 (10.3%)	<0.001	247 (1.3%)	9 (4.2%)	11 (1.8%)	27 (3.2%)	^a^
Witnessed violence or sexual abuse	758 (4.0%)	27 (12.7%)	45 (7.3%)	72 (8.5%)	<0.001	883 (4.7%)	18 (8.5%)	50 (8.1%)	87 (10.3%)	<0.001	92 (0.5%)	1 (0.5%)	8 (1.3%)	5 (0.6%)	^a^
Another frightening, dangerous or violent event	528 (2.8%)	8 (3.8%)	27 (4.4%)	48 (5.7%)	<0.001	901 (4.8%)	8 (3.8%)	40 (6.5%)	103 (12.2%)	<0.001	77 (0.4%)	0 (0.0%)	3 (0.5%)	10 (1.2%)	^a^
Painful medical treatment	629 (3.4%)	7 (3.3%)	28 (4.5%)	49 (5.8%)	<0.001	1247 (6.7%)	24 (11.3%)	48 (7.8%)	85 (10.1%)	<0.001	153 (0.8%)	3 (1.4%)	5 (0.8%)	17 (2.0%)	^a^
Serious illness or accident of a loved one	798 (4.3%)	12 (5.7%)	29 (4.7%)	50 (6.0%)	0.090	5451 (29.2%)	84 (39.6%)	210 (34.1%)	225 (26.9%)	<0.001	1014 (5.4%)	14 (6.6%)	38 (6.2%)	61 (7.3%)	0.105
Childhood neglect	1178 (6.2%)	39 (18.3%)	69 (11.1%)	127 (14.8%)	<0.001										
At least one PTE	6400 (33.8%)	127 (59.6%)	318 (51.2%)	394 (45.8%)	<0.001	9400 (49.8%)	141 (66.2%)	380 (61.4%)	487 (57.0%)	<0.001	1769 (9.4%)	25 (11.7%)	78 (12.6%)	121 (14.2%)	<0.001
Mean number PTEs (SD)	0.53 (0.93)	1.07 (1.21)	0.92 (1.22)	0.88 (1.26)	<0.001	0.80 (1.03)	1.28 (1.35)	1.14 (1.25)	1.12 (1.35)	<0.001	0.11 (0.35)	0.15 (0.45)	0.15 (0.41)	0.18 (0.54)	<0.001

a Not calculated because at least one cell had expected frequencies <5.

### Prevalence of PTEs and education

At least one PTE was reported by 59.1% of the participants with primary and some secondary education, 66.4% of individuals with upper secondary education, 70.3% of the participants with short tertiary education and 72% of the participants with long tertiary education (*p*<0.001). Participants with upper secondary and tertiary education reported significantly more PTEs (upper secondary: *M*=1.46, *SD*=1.65; tertiary, short: *M*=1.64, *SD*=1.77; tertiary, long: *M*=1.67, *SD*=1.72) than participants with primary and partly secondary education (*M*=1.22, *SD*=1.50; *p*<0.001). Table V presents the prevalence of PTEs in the four educational groups for the three time intervals. For most PTEs before and after the age of 18 and for two PTEs during the previous year, significant group differences were observed. Except for another frightening, dangerous or violent event before the age of 18, which was reported significantly more often by participants with primary education, upper secondary and tertiary education were associated with more reported exposure to the specific PTEs (ORs are displayed in Supplemental Table SIV). Combining the two groups with tertiary education to increase statistical power to detect group differences on PTE exposure in the previous year led to only minor changes in the results. Specifically, the differences between educational groups for having witnessed violence or sexual abuse became statistically significant (*p*=0.026).

**Table V.** Prevalence of PTEs by educational level.

	Before the age of 18					After the age of 18					Previous year				
	Primary	Secondary	Tertiary, short	Tertiary, long	*p*	Primary	Secondary	Tertiary, short	Tertiary, long	*p*	Primary	Secondary	Tertiary, short	Tertiary, long	*p*
	*n* (%)	*n* (%)	*n* (%)	*n* (%)		*n* (%)	*n* (%)	*n*(%)	*n* (%)		*n* (%)	*n* (%)	*n* (%)	*n* (%)	
Serious illness or accident	208 (4.5%)	284 (5.1%)	248 (6.3%)	341 (5.6%)	0.002	831 (18.2%)	995 (17.7%)	746 (19.0%)	1022 (16.9%)	0.052	94 (2.1%)	113 (2.0%)	68 (1.7%)	112 (1.9%)	0.658
Violence	184 (4.0%)	327 (5.8%)	258 (6.6%)	434 (7.2%)	<0.001	347 (7.6%)	588 (10.5%)	487(12.4%)	676 (11.2%)	<0.001	14 (0.3%)	27 (0.5%)	15 (0.4%)	37 (0.6%)	0.115
Sexual abuse	248 (5.4%)	401 (7.2%)	318 (8.1%)	515 (8.5%)	<0.001	76 (1.7%)	127 (2.3%)	129 (3.3%)	255 (4.2%)	<0.001	1 (0.0%)	2 (0.0%)	0 (0.0%)	4 (0.1%)	^a^
Bullying	574 (13.6%)	891 (15.9%)	690 (17.6%)	1124 (18.5%)	<0.001	166 (3.6%)	296 (5.3%)	249 (6.3%)	448 (7.4%)	<0.001	32 (0.7%)	70 (1.2%)	66 (1.7%)	124 (2.0%)	<0.001
Witnessed violence or sexual abuse	144 (3.2%)	258 (4.6%)	211 (5.4%)	285 (4.7%)	<0.001	185 (4.1%)	286 (5.1%)	233 (5.9%)	323 (5.3%)	<0.001	22 (0.5%)	41 (0.7%)	16 (0.4%)	25 (0.4%)	0.063
Another frightening, dangerous or violent event	191 (4.2%)	154 (2.7%)	98 (2.5%)	142 (2.3%)	<.001	153 (3.4%)	263 (4.7%)	252 (6.4%)	375 (6.2%)	<.001	15 (0.3%)	26 (0.5%)	19 (0.5%)	31 (0.5%)	0.551
Painful medical treatment	109 (2.4%)	165 (3.0%)	146 (3.7%)	284 (4.7%)	<0.001	272 (6.0%)	371 (6.6%)	316 (8.0%)	430 (7.1%)	0.002	35 (0.7%)	41 (0.7%)	46 (1.2%)	55 (0.9%)	0.120
Serious illness or accident of a loved one	118 (2.6%)	237 (4.2%)	179 (4.6%)	351 (5.8%)	<0.001	1136 (25.3%)	1624 (29.1%)	1199 (30.6%)	1947 (32.2%)	<0.001	201 (4.5%)	330 (5.9%)	220 (5.6%)	364 (6.0%)	0.003
Childhood neglect	265 (5.7)	357 (6.3%)	299 (7.6%)	472 (7.8%)	<0.001										
At least one PTE	1327 (28.6%)	1920 (33.9%)	1487 (37.6%)	2419 (39.8%)	<0.001	2043 (44.5%)	2820 (50.1%)	2110 (53.5%)	3317 (54.6%)	<0.001	360 (7.8%)	568 (10.1%)	383 (9.7%)	663 (10.9%)	<0.001
Mean number PTEs (SD)	0.44 (0.87)	0.54 (0.95)	0.61 (1.02)	0.65 (1.01)	<0.001	0.69 (0.96)	0.81 (1.05)	0.91 (1.15)	0.90 (1.08)	<0.001	0.09 (0.33)	0.11 (0.38)	0.11 (0.38)	0.12 (0.38)	<0.001

a Not calculated because at least one cell had expected frequencies <5.

## Discussion

The purpose of the present study was to examine the prevalence of PTEs in the Tromsø 7 study. Overall, 67% of the participants reported at least one PTE during their lifetime. Female sex, younger age, indigenous and immigrant ethnicities and higher education were associated with an increased likelihood of having experienced at least one PTE and a higher number of total PTEs.

The lifetime exposure rate of PTEs found in the present study is similar to the international average of 70.4% [5]. Compared to previous estimates of the prevalence of PTEs in Norway, a considerably higher percentage of individuals reporting PTEs was found than in the studies by Amstadter et al. (26%) [6] and Lassemo et al. (25.9% for men and 20.6% for women, respectively) [7]. However, there was a lower prevalence compared to Heir et al.’s study (85% and 86% for men and women, respectively) [8]. The varying estimates of PTE exposure in Norway may be due to differences in study characteristics, such as sample demographics (e.g. age range, sex distribution, geographical region) and assessment of PTEs. For example, unlike previous studies, the participants in the current study resided solely in Northern Norway, and the age range was restricted to ⩾40 years. Further, participants were asked to consider if a given PTE occurred in childhood, adulthood or during the previous year, which may have facilitated the recall of these events.

In line with previous findings [6,23,24], men were more likely to have experienced violence and a life-threatening illness or serious accident, whereas women were at much higher risk of exposure to sexual abuse or witnessing another’s sexual abuse. Further, women more often reported childhood neglect, painful treatment in hospital and a life-threatening illness or accident of a loved one. Regardless of sex differences in the total exposure to PTEs, findings suggest that women have a higher risk of experiencing PTEs that function as risk factors for the development of posttraumatic stress disorder [8,25].

With respect to the associations of PTEs with age, a decrease in most PTEs with increasing age was found. Although it is reasonable to expect an increase with longevity, which has also been observed in some investigations [23,26], similar trends have been reported in several other studies [7,27,28]. Different explanations for this observation have been discussed, including cohort effects, prolonged recall period, recall bias due to age-related cognitive decline and selective mortality [27]. It is further possible that elderly who have not been exposed to PTEs are more likely to participate in research. Differences between cohorts in the perception of the seriousness of an event and personal sensitivity may also affect the reporting of PTEs [8]. In the present investigation, a notable exception to the age effect was exposure to another frightening, dangerous or violent event (e.g. a natural disaster, war, terror attack, being held captive), which was significantly more often reported by participants aged >70 years. It is conceivable that the oldest participants related this PTE to their experience of World War II, resulting in an elevated prevalence due to a cohort effect.

Consistent with previous studies on the association of PTEs with ethnicity and minority status [28], Sami/Kven and participants with immigrant ethnicities were more likely to have experienced PTEs. The findings replicate the results from earlier investigations showing that Sami are more exposed to violence and bullying than non-Sami individuals [9,29]. Also, the findings suggests that Sami/Kven are more likely to have experienced, during adulthood, painful hospital treatment and a life-threatening illness or serious accident of a loved one.

In contrast to reviews that concluded that there is a greater risk of PTE exposure in individuals with lower educational level [28], a positive relationship between education and PTE prevalence in childhood and adulthood was observed in the current sample, with only a few exceptions. Although a positive relationship between education and PTEs has occasionally been reported [5], the strength of the associations in the present study is striking and difficult to explain. It can be speculated that individuals with higher education are more aware of PTEs and therefore report these events to a higher degree than individuals with lower education, leading to the observed differences in the reported PTEs. It is also possible that individuals with lower education who have been exposed to PTEs are underrepresented in Tromsø 7.

The study has several limitations that need to be taken into consideration when interpreting the results. Despite the large sample size, the representativeness of the population in terms of age, education and ethnicity is unclear, and response-rate bias may have affected the results. In the assessment of PTEs, different PTEs were collapsed into one question (e.g. life-threatening illness and serious accident), and that the degree of traumatisation for each PTEs was not examined. The response options ‘after 18’ and ‘previous year’ were not mutually exclusive, which may have led participants to select both options for a PTE in the previous year. Low counts for some demographic categories resulted in reduced statistical power to detect group differences. Finally, interactions between demographic variables (e.g. age and education) were not examined.

In conclusion, exposure to PTEs is common in the Tromsø 7 population, with about two thirds of the participants reporting at least one PTE during their lifetime. Higher overall prevalence of PTEs was associated with female sex, younger age, indigenous or immigrant ethnicities and higher education, and exposure to specific PTEs varied with sex. Our findings will be relevant for researchers investigating the role of isolated and multiple PTEs in childhood and adulthood for physical and mental health variables collected in the Tromsø study.

## Supplemental Material

sj-docx-1-sjp-10.1177_14034948211051511 – Supplemental material for The prevalence of potentially traumatic events in the seventh survey of the population-based Tromsø study (Tromsø 7) Click here for additional data file. Supplemental material, sj-docx-1-sjp-10.1177_14034948211051511 for The prevalence of potentially traumatic events in the seventh survey of the population-based Tromsø study (Tromsø 7) by Jens C. Thimm, Kamilla Rognmo, Marte Rye, Anna Margrete Flåm, Eva Therese Næss, Ingunn Skre and Catharina E. A. Wang in Scandinavian Journal of Public Health
