# Supplementary material for: The prevalence of potentially traumatic events in the seventh survey of the population-based Tromsø study (Tromsø 7)
Source: Scand J Public Health. 2021 Oct 20;51(7):1050–60. doi: 10.1177/14034948211051511 (PMC10599077; doi:10.1177/14034948211051511)
Supplement: sj-docx-1-sjp-10.1177_14034948211051511 – Supplemental material for The prevalence of potentially traumatic events in the seventh survey of the population-based Tromsø study (Tromsø 7) [file sj-docx-1-sjp-10.1177_14034948211051511.docx]

**Online supplementary material**

Table S1. Odd ratios and 95% confidence intervals for gender (females as reference group)

|  | Before age 18 | After age 18 | Previous year |
| --- | --- | --- | --- |
|  | *OR* (95% CI) | *OR* (95% CI) | *OR* (95% CI) |
| Serious illness or accident | 1.26 (1.12, 1.43) | 1.47 (1.37, 1.58) | 1.13 (0.92, 1.39) |
| Violence | 1.71 (1.51, 1.93) | 1.39 (1.27, 1.52) | 1.03 (0.67, 1.58) |
| Sexual abuse | 0.25 (0.22, 0.28) | 0.10 (0.07, 0.13) |  |
| Bullying | 1.11 (1.03, 1.20) | 0.65 (0.57, 0.74) | 0.62 (0.48, 0.79) |
| Witnessed violence or sexual abuse | 0.78 (0.68, 0.89) | 0.91 (0.80, 1.03) | 0.68 (0.45, 1.02) |
| Another frightening, dangerous, or violent event | 0.76 (0.64, 0.90) | 1.77 (1.55, 2.01) | 0.79 (0.50, 1.22) |
| Painful medical treatment | 0.88 (0.76, 1.03) | 0.83 (0.74, 0.93) | 0.90 (0.66, 1.22) |
| Serious illness or accident of a loved one | 0.96 (0.83, 1.10) | 0.69 (0.65, 0.73) | 0.77 (0.68, 0.87) |
| Childhood neglect | 0.64 (0.57, 0.71) |  |  |
| At least one PTE | 0.89 (0.85, 0.95) | 0.86 (0.81, 0.91) | 0.85 (0.77, 0.93) |

Note. CI = confidence interval; OR = odds ratio.

Table S2. Odd ratios and 95% confidence intervals for age groups (age group 40-49 years as reference group)

|  | Before age 18 | | | | | After age 18 | | | | | Previous year | | | | |
| --- | --- | --- | --- | --- | --- | --- | --- | --- | --- | --- | --- | --- | --- | --- | --- |
|  | 40-49 | 50-59 | 60-69 | 70-79 | 80-99 | 40-49 | 50-59 | 60-69 | 70-79 | 80-99 | 40-49 | 50-59 | 60-69 | 70-79 | 80-99 |
|  | ref. | *OR*  (95% CI) | *OR*  (95% CI) | *OR* (95% CI) | *OR*  (95% CI) | ref. | *OR*  (95% CI) | *OR*  (95% CI) | *OR*  (95% CI) | *OR*  (95% CI) | ref. | *OR*  (95% CI) | *OR*  (95% CI) | *OR*  (95% CI) | *OR*  (95% CI) |
| Serious illness or accident |  | 0.81  (0.70, 0.94) | 0.56  (0.47, 0.66) | 0.41  (0.31, 0.52) | 0.47  (0.30, 0.71) |  | 1.39  (1.26, 1.53) | 1.40  (1.27, 1.54) | 1.53  1.36, 1.72) | 1.49  (1.22, 1.81) |  | 1.34  (1.02, 1.76) | 1.51  (1.15, 2.00) | 1.51  (1.08, 2.01) | 1.08  (0.54, 1.94) |
| Violence |  | 0.70  (0.61, 0.80) | 0.36  (0.31, 0.43) | 0.19  (0.14, 0.25) | 0.12  (0.05, 0.22) |  | 0.84  (0.76, 0.93) | 0.53  (0.46, 0.59) | 0.23  (0.18, 0.28) | 0.19  (0.12, 0.29) |  | 0.52  (0.32, 0.83) | 0.39  (0.21, 0.67) | 0.05  (0.00, 0.22) | a |
| Sexual abuse |  | 0.92  (0.81, 1.04) | 0.65  (0.56, 0.75) | 0.33  0.26, 0.41) | 0.19  (0.10, 0.31) |  | 0.83  (0.68, 1.01) | 0.63  (0.50, 0.78) | 0.32  (0.21, 0.45) | 0.18  (0.07, 0.40) |  | 0.15  (0.01, 0.86) | a | a | a |
| Bullying |  | 0.72  (0.65, 0.78) | 0.41  (0.37, 0.45) | 0.24  (0.20, 0.28) | 0.17  (0.11, 0.23) |  | 0.88  (0.76, 1.01) | 0.63  (0.54, 0.74) | 0.39  (0.30, 0.50) | 0.25  (0.13, 0.41) |  | 1.07  (0.83, 1.38) | 0.44  (0.31, 0.62) | 0.20  (0.10, 0.36) | a |
| Witnessed violence or sexual abuse |  | 0.69  (0.59, 0.80) | 0.38  (0.31, 0.46) | 0.16  (0.11, 0.23) | 0.14  (0.06, 0.27) |  | 0.93  (0.80, 1.08) | 0.55  (0.46, 0.66) | 0.41  (0.31, 0.52) | 0.32  (0.18, 0.53) |  | 0.69  (0.44, 1.06) | 0.34  (0.18, 0.60) | 0.33  (0.14, 0.69) | 0.36  (0.06, 1.16) |
| Another frightening, dangerous, or violent event |  | 0.62  (0.45, 0.83) | 0.64  (0.46, 0.87) | 4.30  (3.41, 5.45) | 18.36  (14.31, 23.64) |  | 1.08  (0.93, 1.26) | 0.69  (0.58, 0.82) | 0.54  (0.42, 0.68) | 0.59  (0.37, 0.87) |  | 0.90  (0.56, 1.44) | 0.29  (0.13, 0.57) | 0.38  (0.14, 0.82) | 0.95  (0.28, 2.36) |
| Painful medical treatment |  | 1.07  (0.89, 1.28) | 0.91  (0.74, 1.11) | 0.63  (0.47, 0.84) | 0.47  (0.25, 0.81) |  | 1.03  (0.90, 1.18) | 0.80  (0.69, 0.93) | 0.83  (0.69, 1.00) | 0.74  (0.51, 1.02) |  | 0.88  (0.61, 1.27) | 0.81  (0.54, 1.20) | 0.59  (0.32, 1.00) | 1.05  (0.44, 2.14) |
| Serious illness or accident of a loved one |  | 0.70  (0.60, 0.82) | 0.50  (0.42, 0.60) | 0.33  (0.24, 0.43) | 0.32  (0.18, 0.53) |  | 1.12  (1.04, 1.21) | 0.95  (0.87, 1.03) | 0.82  (0.74, 0.91) | 0.75  (0.62, 0.90) |  | 0.88  (0.76, 1.03) | 0.72  (0.61, 0.85) | 0.81  (0.66, 0.99) | 0.53  (0.33, 0.79) |
| Childhood neglect |  | 0.91  (0.80, 1.04) | 0.56  (0.48, 0.65) | 0.45  (0.36, 0.56) | 0.53  (0.37, 0.75) |  |  |  |  |  |  |  |  |  |  |
| At least one PTE |  | 0.74  (0.69, 0.79) | 0.47  (0.43, 0.50) | 0.38  (0.34, 0.42) | 0.64  (0.55, 0.76) |  | 1.08  (1.01, 1.16) | 0.85  (0.79, 0.92) | 0.75  0.68, 0.82) | 0.69  (0.59, 0.80) |  | 0.94  (0.84, 1.06) | 0.73  (0.64, 0.82) | 0.73  (0.62, 0.86) | 0.54  (0.39, 0.74) |

Note. ^a^ Category excluded from analysis due to zero count. CI = confidence interval; OR = odds ratio; ref. = reference group.

Table S3. Odd ratios and 95% confidence intervals for ethnic groups (Norwegian ethnicity as reference group)

|  | Before age 18 | | | | After age 18 | | | | Previous year | | | |
| --- | --- | --- | --- | --- | --- | --- | --- | --- | --- | --- | --- | --- |
|  | Norw. | Sami/ Kven | Norw.-Sami/ Kven | Other | Norw. | Sami/ Kven | Norw.-Sami/ Kven | Other | Norw. | Sami/ Kven | Norw.-Sami/ Kven | Other |
|  | ref. | *OR*  (95% CI) | *OR*  (95% CI) | *OR*  (95% CI) | ref. | *OR*  (95% CI) | *OR*  (95% CI) | *OR*  (95% CI) | ref. | *OR*  (95% CI) | *OR*  (95% CI) | *OR*  (95% CI) |
| Serious illness or accident |  | 2.04  (1.26, 3.14) | 2.01  (1.51, 2.62) | 1.40  (1.06, 1.82) |  | 1.26  (0.90, 1.74) | 1.46  (1.21, 1.76) | 1.14  (0.95, 1.35) |  | 0.78  (0.19, 2.06) | 1.83  (1.12, 2.82) | 1.80  (1.18, 2.63) |
| Violence |  | 2.54  (1.66, 3.76) | 1.91  (1.44, 2.49) | 2.42  (1.94, 2.99) |  | 2.80  (2.01, 3.84) | 1.93  (1.55, 2.38) | 1.90  (1.57, 2.28) |  | 2.34  (0.38, 7.51) | 1.60  (0.49, 3.87) | 2.94  (1.42, 5.44) |
| Sexual abuse |  | 1.96  (1.27, 2.89) | 2.41  (1.91, 3.01) | 1.34  (1.05, 1.70) |  | 2.63  (1.45, 4.39) | 2.51  (1.77, 3.47) | 2.09  (1.51, 2.82) |  | a | 6.09  (0.32, 37.8) | 8.88  (1.27, 41.2) |
| Bullying |  | 2.15  (1.58, 2.89) | 1.93  (1.60, 2.31) | 1.43  (1.20, 1.69) |  | 3.11  (2.07, 4.51) | 2.18  (1.66, 2.81) | 2.08  (1.64, 2.60) |  | 3.31  (1.56, 6.18) | 1.36  (0.70, 2.39) | 2.47  (1.61, 3.63) |
| Witnessed violence or sexual abuse |  | 3.47  (2.25, 5.13) | 1.87  (1.35, 2.52) | 2.21  (1.71, 2.83) |  | 1.88  (1.11, 2.97) | 1.78  (1.31, 2.37) | 2.32  (1.83, 2.91) |  | 0.96  (0.05, 4.35) | 2.66  (1.18, 5.17) | 1.21  (0.42, 2.69) |
| Another frightening, dangerous, or violent event |  | 1.36  (0.61, 2.59) | 1.58  (1.04, 2.30) | 2.08  (1.51, 2.78) |  | 0.78  (0.35, 1.48) | 1.37  (0.97, 1.88) | 2.75  (2.20, 3.40) |  | a | 1.18  (0.29, 3.18) | 2.90  (1.40, 5.36) |
| Painful medical treatment |  | 0.98  (0.42, 1.94) | 1.36  (0.91, 1.97) | 1.78  (1.30, 2.38) |  | 1.79  (1.14, 2.69) | 1.18  (0.86, 1.57) | 1.58  (1.24, 1.98) |  | 1.74  (0.43, 4.64) | 0.99  (0.35, 2.18) | 2.51  (1.46, 4.03) |
| Serious illness or accident of a loved one |  | 1.34  (0.71, 2.31) | 1.11  (0.74, 1.59) | 1.42  (1.04, 1.89) |  | 1.59  (1.20, 2.09) | 1.25  (1.06, 1.48) | 0.89  (0.76, 1.04) |  | 1.23  (0.68, 2.04) | 1.14  (0.81, 1.57) | 1.37  (1.03, 1.77) |
| Childhood neglect |  | 3.37  (2.34, 4.74) | 1.89  (1.45, 2.42) | 2.62  (2.12, 3.18) |  |  |  |  |  |  |  |  |
| At least one PTE |  | 2.89  (2.20, 3.82) | 2.06  (1.75, 2.41) | 1.66  (1.44, 1.90) |  | 1.97  (1.49, 2.63) | 1.60  (1.36, 1.89) | 1.34  (1.16, 1.53) |  | 1.28  (0.82, 1.92) | 1.39  (1.09, 1.76) | 1.59  (1.30, 1.94) |

Note. ^a^ Category excluded from analysis due to zero count. CI = confidence interval; Norw. = Norwegian; OR = odds ratio; ref. = reference group.

Table S4. Odd ratios and 95% confidence intervals for education groups (primary education as reference group)

|  | Before age 18 | | | | After age 18 | | | | Previous year | | | |
| --- | --- | --- | --- | --- | --- | --- | --- | --- | --- | --- | --- | --- |
|  | Primary | Secon-dary | Tertiary, short | Tertiary, long | Primary | Secon-dary | Tertiary, short | Tertiary, long | Primary | Secon-dary | Tertiary, short | Tertiary, long |
|  | ref. | *OR*  (95% CI) | *OR*  (95% CI) | *OR*  (95% CI) | ref. | *OR*  (95% CI) | *OR*  (95% CI) | *OR*  (95% CI) | ref. | *OR*  (95% CI) | *OR*  (95% CI) | *OR*  (95% CI) |
| Serious illness or accident |  | 1.12  (0.93, 1.34) | 1.41  (1.17, 1.71) | 1.25  (1.05, 1.50) |  | 0.97  (0.88, 1.07) | 1.06  (0.95, 1.18) | 0.91  (0.83, 1.01) |  | 0.98  (0.74, 1.29) | 0.84  (0.61, 1.15) | 0.90  (0.68, 1.19) |
| Violence |  | 1.47  (1.22, 1.78) | 1.67  (1.38, 2.03) | 1.84  (1.54, 2.20) |  | 1.42  (1.24, 1.64) | 1.72  (1.49, 1.99) | 1.53  (1.34, 1.75) |  | 1.57  (0.84, 3.09) | 1.24  (0.60, 2.61) | 2.00  (1.10, 3.83) |
| Sexual abuse |  | 1.34  (1.14, 1.58) | 1.53  (1.29, 1.82) | 1.62  (1.38, 1.89) |  | 1.37  (1.03, 1.83) | 2.00  (1.51, 2.68) | 2.60  (2.01, 3.38) |  | 1.63  (0.16, 35.04) | a | 3.02  (0.45, 59.00) |
| Bullying |  | 1.31  (1.17, 1.47) | 1.48  (1.31, 1.67) | 1.58  (1.42, 1.76) |  | 1.47  (1.22, 1.79) | 1.79  (1.46, 2.19) | 2.11  (1.76, 2.54) |  | 1.79  (1.19, 2.75) | 2.41  (1.59, 3,73) | 2.95  (2.03, 4.44) |
| Witnessed violence or sexual abuse |  | 1.48  (1.20, 1.82) | 1.74  (1.40, 2.16) | 1.51  (1.24, 1.86) |  | 1.27  (1.05, 1.54) | 1.49  (1.22, 1.82) | 1.33  (1.11, 1.61) |  | 1.52  (0.91, 2.59) | 0.84  (0.43, 1.60) | 0.86  (0.48, 1.53) |
| Another frightening, dangerous, or violent event |  | 0.64  (0.52, 0.80) | 0.58  (0.45, 0.75) | 0.55  (0.44, 0.68) |  | 1.41  (1.16, 1.74) | 1.97  (1.61, 2.43) | 1.90  (1.57, 2.30) |  | 1.41  (0.75, 2.72) | 1.47  (0.75, 2.94) | 1.56  (0.85, 2.96) |
| Painful medical treatment |  | 1.23  (0.96, 1.58) | 1.56  (1.22, 2.01) | 1.99  (1.60, 2.50) |  | 1.11  (0.94, 1.31) | 1.37  (1.16, 1.62) | 1.20  (1.02, 1.40) |  | 0.95  (0.60, 1.50) | 1.52  (0.98, 2.38) | 1.18  (0.77, 1.81) |
| Serious illness or accident of a loved one |  | 1.65  (1.32, 2.07) | 1.78  (1.41, 2.26) | 2.29  (1.86, 2.84) |  | 1.21  (1.11, 1.33) | 1.31  (1.19, 1.44) | 1.41  (1.29, 1.54) |  | 1.34  (1.12, 1.61) | 1.27  (1.05, 1.55) | 1.37  (1.15, 1.64) |
| Childhood neglect |  | 1.12  (0.95, 1.31) | 1.35  (1.14, 1.61) | 1.39  (1.19, 1.63) |  |  |  |  |  |  |  |  |
| At least one PTE |  | 1.29  (1.18, 1.40) | 1.51  1.38, 1.65() | 1.65  (1.52, 1.79) |  | 1.25  (1.16, 1.35) | 1.44  (1.32, 1.57) | 1.50  (1.39, 1.62) |  | 1.32  (1.15, 1.52) | 1.27  (1.09, 1.47) | 1.44  (1.26, 1.65) |

Note. ^a^ Category excluded from analysis due to zero count. CI = confidence interval; OR = odds ratio; ref. = reference group
